# Signature of click chemistry in advanced techniques for cancer therapeutics

**DOI:** 10.1039/d5ra01196e

**Published:** 2025-04-04

**Authors:** Sherif Shaban Ragab

**Affiliations:** a Photochemistry Department, Chemical Industries Research Institute, National Research Centre El-Buhouth St, P.O. 12622, Dokki Giza Egypt she2rifx@yahoo.com

## Abstract

Click chemistry has made a revolution in the field of chemical biology owing to its high efficiency, specificity, and mild reaction conditions. The copper(i)-catalyzed azide–alkyne cycloaddition (CuAAC) and strain-promoted [3 + 2] azide–alkyne cycloaddition (SPAAC) stand out as the most popular click reactions that construct a stable triazole ring by reacting an azide with an alkyne. These two reactions represent an ideal choice for biological applications due to its specificity, reliability, and biocompatibility. As a powerful modular synthetic approach for creating new molecular entities, it has seen increasing use in anticancer drug discovery. The present “state of the art” focuses mainly on the signature of click chemistry (CuAAC and SPAAC) in advanced techniques for cancer therapeutics, which includes cancer immunotherapy, antibody–drug conjugates, development of proteolysis-targeting chimeras, targeted dual-agent combination therapy for cancer, exosome modification for cancer therapy, and photodynamic therapy (PDT).

## Abstract 2

This review focuses mainly on the signature of click chemistry (CuAAC and SPAAC) in advanced techniques for cancer therapeutics.

## Graphical abstract

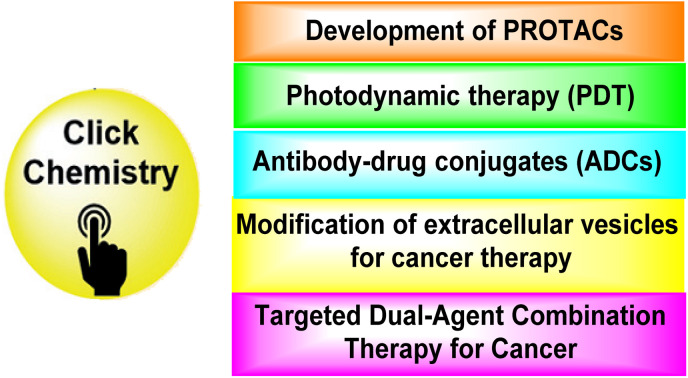

## 1. Introduction

The concept of click chemistry gained global recognition when the Royal Swedish Academy of Sciences awarded the 2022 Nobel Prize in Chemistry to Bertozzi, Meldal, and Sharpless on October 5, 2022.^1^ This honor acknowledged their work in “click chemistry and bioorthogonal chemistry.”

Click chemistry is a molecular assembly technique comparable to connecting Lego bricks, enabling swift and selective molecule combination to create complex drug compounds. The term “click” means linking molecular species in an easy manner resembling the clicking of the two pieces of a seat belt buckle.^2^ Synthetic chemistry and biology extensively employ click approaches, which demonstrate remarkable adaptability while being economical in atom usage and, in certain instances, biocompatible.^3^

**Fig. 1:**
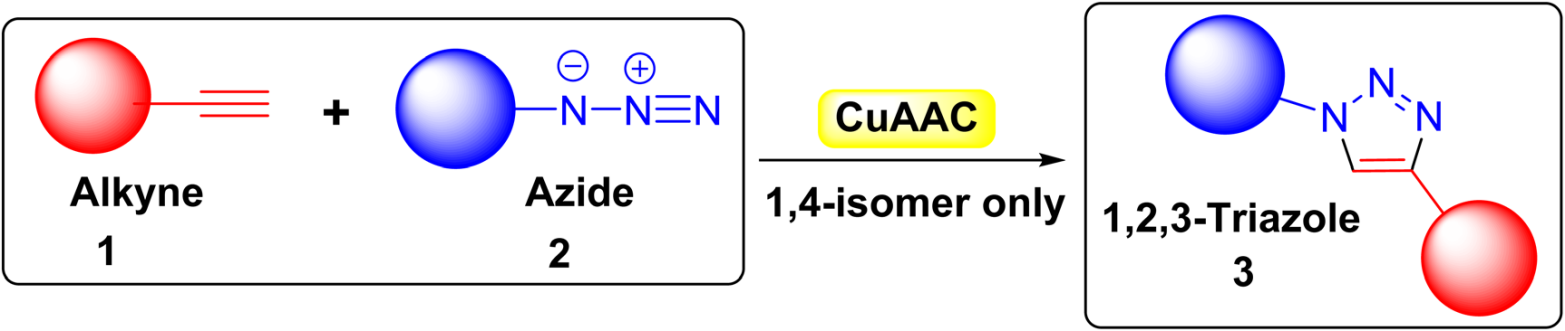
Copper(i)-catalyzed [3 + 2] azide–alkyne cycloaddition (CuAAC).

There are many types of click chemistry with numerous applications,^4^ but CuAAC is the most common and is synonymous with click chemistry. CuAAC involves reacting an alkyne 1 with an azide 2 to produce only 1,4-regioisomers of 1,2,3-triazoles 3 (Fig. 1) with near-perfect yield, eliminating the need for additional purification methods such as recrystallization or column chromatography. The ability to combine various alkynes 1 and azides 2 enables rapid creation of extensive compound libraries, with diverse biological activities. Additionally, the reaction can occur in water, leading to widespread use of click chemistry across numerous research fields.^5^

To remove the toxicity of Cu(i) ions, the Bertozzi group developed a novel reaction without using Cu(i) catalyst, called strain-promoted [3 + 2] azide–alkyne cycloaddition (SPAAC), as an alternative to the CuAAC reaction.^6^

Innovative approaches like *in situ* click chemistry have provided some insights into novel methods for creating highly effective enzyme inhibitors. Moreover, click chemistry is utilized in medicinal chemistry for developing agonists, antagonists, and selective ligands in receptor–ligand binding studies for drug discovery.^2^

Bioorthogonal-click chemistry reactions are opening up new avenues for biological innovations. This technique has been successfully employed for site-specific labeling of proteins, glycans, lipids, and cell surfaces within living organisms under physiological conditions. Click-activity-based protein profiling (click-ABPP) platforms facilitate the identification of various disease-relevant enzymes and the development of selective pharmacological probes to disrupt and study these proteins in cellular environments. Activity-based proteomics can offer insights into metabolic and signaling pathways, potentially revealing new approaches for disease diagnosis and treatment.^7^

The versatility of azide labeling and Cu-free click chemistry allows for applications across various areas of chemical biology. SPAAC has been employed to monitor azidosugars, proteins containing azido amino acids, lipids, and specifically labeled proteins, DNA, and RNA in living cells.^8^

The term “cancer” encompasses a group of diseases characterized by the abnormal and uncontrolled growth of cells. It poses a significant threat to human health, ranking second only to cardiovascular disease as a leading cause of morbidity and mortality.^9^

The International Agency for Research on Cancer (IARC) recently estimated 20 million cancer cases and 9.7 million deaths globally in 2022, with projections reaching 35 million by 2050.^10^ Treatment options for cancer patients include surgery, chemotherapy, radiation, hormone therapy, or a combination of these approaches. While surgery and radiation remain primary interventions for accessible tumors, they are limited in their ability to completely eradicate all cancer cells, often necessitating follow-up chemotherapy. Targeted cancer therapies aim to inhibit cancer cell growth and proliferation by interfering with specific molecular targets involved in cancer development and progression.^11^

Current cancer treatments often result in undesirable side effects and drug resistance. Many anti-cancer drugs lack the ability to differentiate between healthy and cancerous cells, leading to toxicity in normal cells. The ineffectiveness of chemotherapy due to drug resistance and non-selective targeting underscores the urgent need for novel chemo-therapeutic agents with improved efficacy, reduced toxicity, and enhanced selectivity.^12^

1,2,3-Triazoles, the primary and most popular product of click reactions, represent a crucial category of nitrogen-containing heterocycles. These compounds can constitute varied non-covalent interactions with proteins, enzymes, and receptors. As a result, triazole derivatives have garnered significant interest due to their therapeutic potential,^13,14^ particularly in anticancer applications.^15^

A comprehensive survey of click chemistry and its signature in advanced techniques for cancer therapeutics would be valuable to a broad audience of researchers in chemistry, biology, and medicine. The current “state of the art” aims to provide a concise overview of recent studies (2020 to 2025) focusing on the signature of click chemistry with its three main complementary types (copper-catalyzed (CuAAC), copper-free (SPACC), and biorthogonal click chemistry) in advanced techniques for cancer therapeutics which include cancer immunotherapy, synthesis of antibody–drug conjugates, development of PROTACs, targeted dual-agent combination therapy for cancer, exosome modification for cancer therapy, and photodynamic therapy (PDT).

## 2. Copper-catalyzed azide–alkyne cycloaddition (CuAAC)

The idea of click chemistry was first coined by K. Barry Sharpless from the Scripps Research Institute two decades ago. Soon after, Sharpless and Morten Meldal from the University of Copenhagen independently developed copper-catalyzed azide–alkyne cycloaddition (CuAAC), which is synonymous and emblematic of click chemistry.^2^

The CuAAC reaction produces 1,4-disubstituted 1,2,3-triazoles with remarkable selectivity and high yields. CuAAC has become a standard method for synthesizing 1,2,3-triazoles, many of which show promising biomedical applications.^14–16^ The CuAAC reaction has also been employed to create triazole-based ligands, and for chemical conjugations in biological labeling.^17^

The reaction's solvent flexibility, spanning from organic to aqueous media, is noteworthy. The most common protocol established by Sharpless and his coworkers utilizes CuSO_4_ and 10 equivalents of sodium ascorbate in an aqueous solvent mixture (water + alcohol such as *t*-BuOH, MeOH, or EtOH). This combination solubilizes the substrate while preserving the advantageous aqueous environment. These aqueous conditions are particularly valuable for both biochemical conjugations and organic syntheses.^18^

**Fig. 2:**
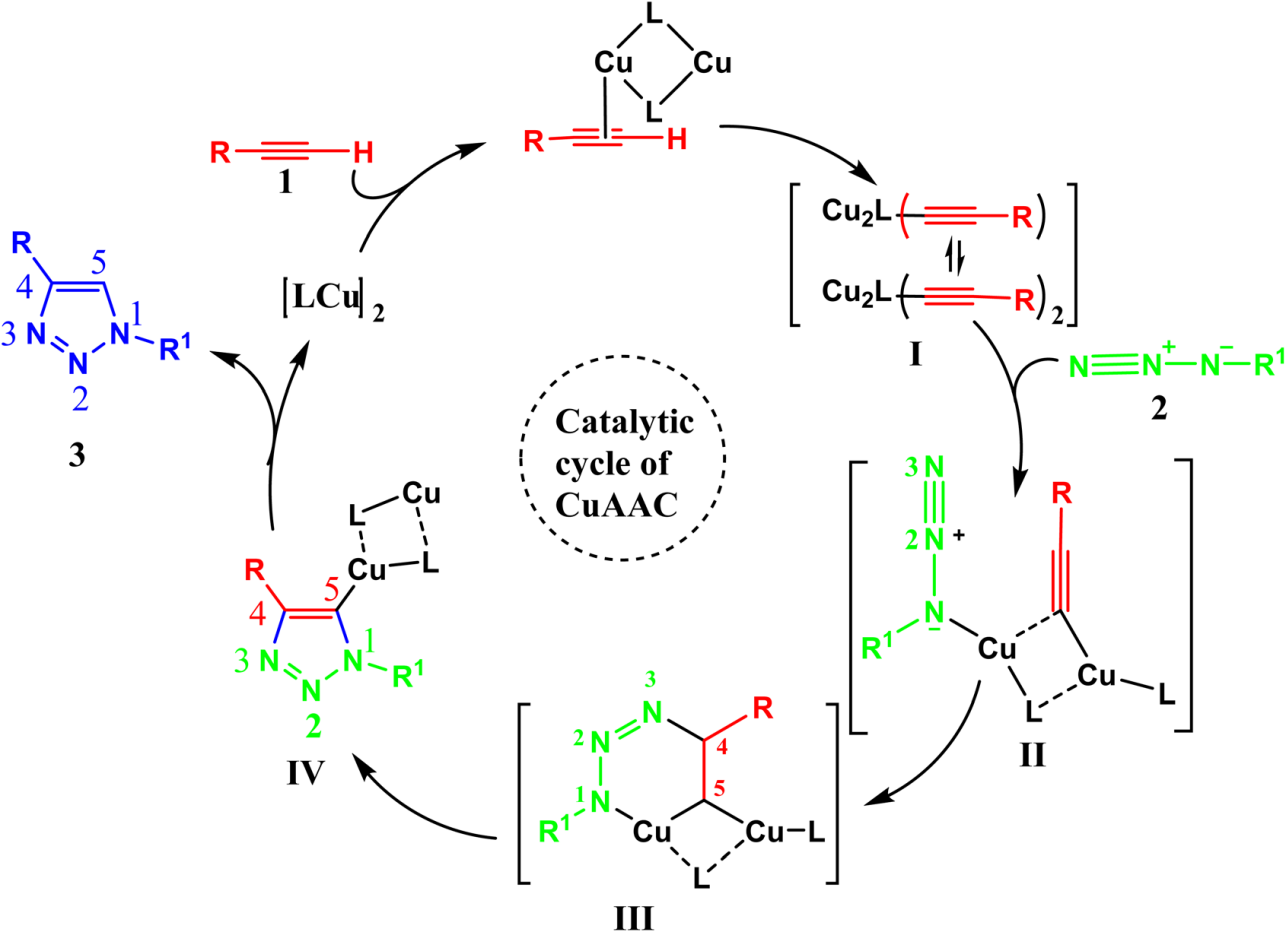
Plausible mechanism of Cu(i)-catalyzed azide–alkyne cycloaddition (CuAAC).^5^

A plausible mechanism^3^ of Cu(i)-catalyzed azide–alkyne cycloaddition (CuAAC) is shown in Fig. 2. Click reaction's catalytic process initiates with the creation of the copper acetylide I intermediate from the Cu–alkyne complex. This intermediate, upon further coordination extension and interaction with azide 2, forms complex II. Subsequently, in complex II, the terminal N-3's nucleophilic properties favor an attack on the C-4 position, resulting in a stable spatial arrangement. This leads to the transformation of complex II into complex III. The rearrangement continues through a six-membered transition state, where the lone pair of N-1 in the metallacycle attacks C-5, producing a more compact and efficient ring-structured species IV.

The catalytic cycle concludes with the protonation of intermediate reactive species IV, facilitated by a base or solvent. This process yields the target 1,4-disubstituted-1,2,3-triazole product 3 and simultaneously triggers the effortless detachment of the copper complex. The liberated Cu-system then re-engages with terminal alkyne 1, forming acetylide complex I and perpetuating the catalytic sequence.^3^

## 3. Strain-promoted azide–alkyne [3 + 2] cycloaddition

The Bertozzi group addressed the problem concerning the toxicity of Cu(i) ions by developing a novel reaction without using Cu(i) catalyst, called strain-promoted [3 + 2] azide–alkyne cycloaddition (SPAAC), as an alternative to CuAAC reaction.^6^

**Fig. 3:**
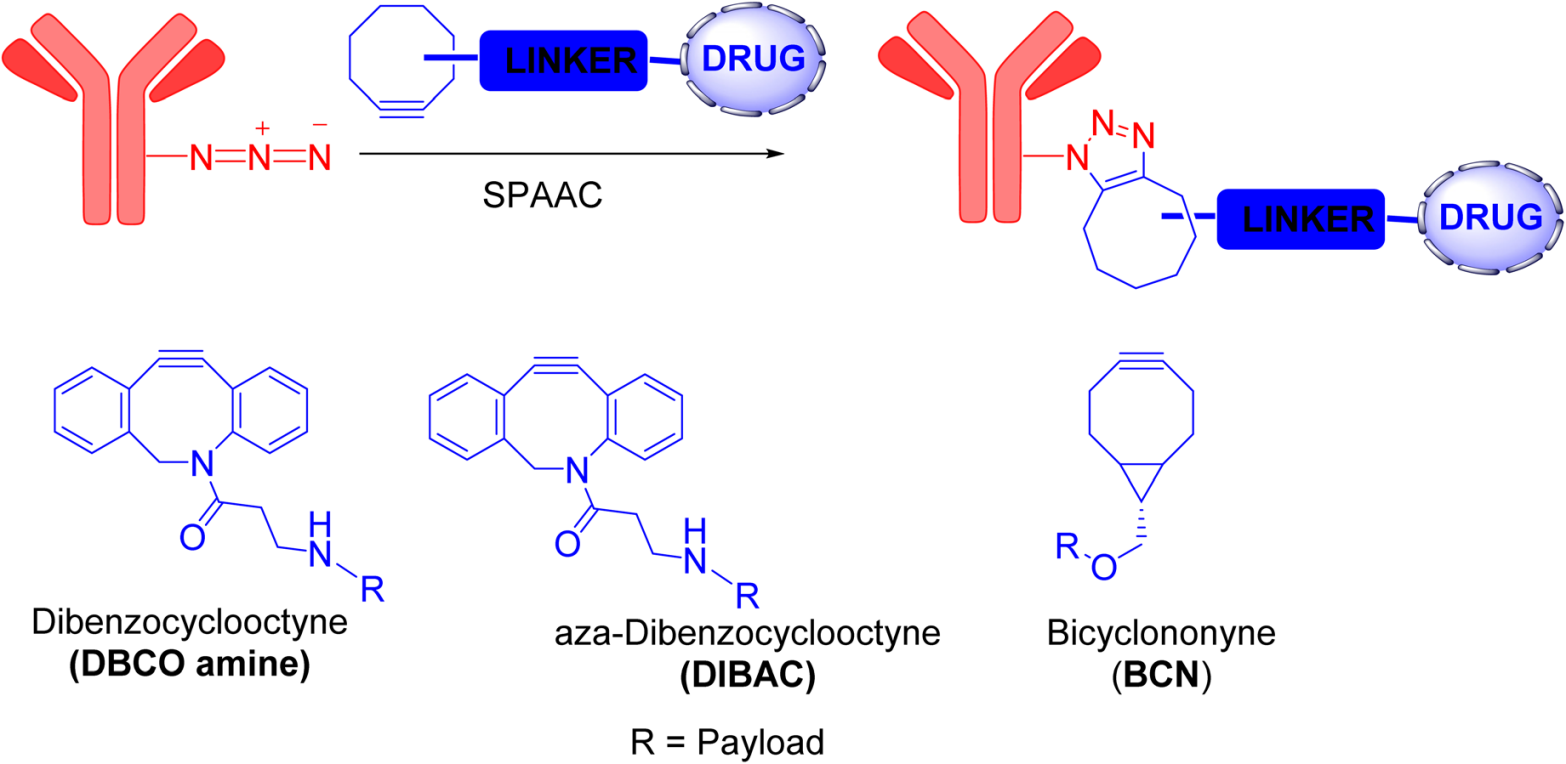
SPAAC reaction and the cycloalkynes used for this strategy.^19^

The concept behind this reaction is related to the alleviation of ring strain in cycloalkynes. When these compounds interact with organic azides, a favorable transformation from a strained ring to a fused ring occurs, forming triazoles with sp^2^-hybridized carbon atoms. Although the reaction was quick enough to be classified as a click reaction, it remained significantly slower than its CuAAC counterpart. Numerous efforts to enhance the SPAAC reaction have been made, primarily by modifying the cycloalkyne structure, as it directly influences the reaction rate. The cycloalkyne's reactivity, lipophilicity, and size all impact the SPAAC reaction rate; consequently, novel and improved cycloalkynes were developed.^4^ Notable examples include dibenzoannulated cyclooctyne (DIBO), dibenzocyclooctyne (DBCO) amine, aza-dibenzocyclooctyne (DIBAC), and bicyclononyne (BCN) (Fig. 3).^19^ The increased ring strain in these new compounds caused by multiple sp^2^-hybridized carbon atoms significantly improve the SPAAC reaction rate. In fact, SPAAC is rapid, specific, bioorthogonal, and safer than CuAAC.

## 4. Bioorthogonal click chemistry

There is an urgent need for innovative strategies to selectively release potent cytotoxic drugs in tumor regions in an “on-demand” manner.^20^ To address this challenge, the concept of bioorthogonal chemistry, which is compatible with and orthogonal to biological systems, has garnered significant attention.^21–23^ The primary advantage of this reaction is its ability to occur in biological environments without interfering with innate biochemical processes. Moreover, it demonstrates high chemical selectivity for molecules commonly found in cells.^24^

The prevailing concept has principally focused on the “bond formation” reaction between two mutually reactive components, typically involving a two-step process.

Initially, a specific component (in particular, an azide motif) is incorporated into target biomolecules through chemical-, metabolic-, or genetic methods. Subsequently, a corresponding probe (*e.g.*, a DBCO or BCN group) with desired functional modifications is externally introduced.^25^ The excellent affinity between both bioorthogonal probes allows for efficient linking of functional components and target molecules *in vivo*. This process is implemented by the most renowned click reaction (CuAAC) owing to the superior bio-orthogonality of both azide and alkyne motifs participating in the targeted reaction.

### 4.1. Bioorthogonal click chemistry in cancer immunotherapy

**Fig. 4:**
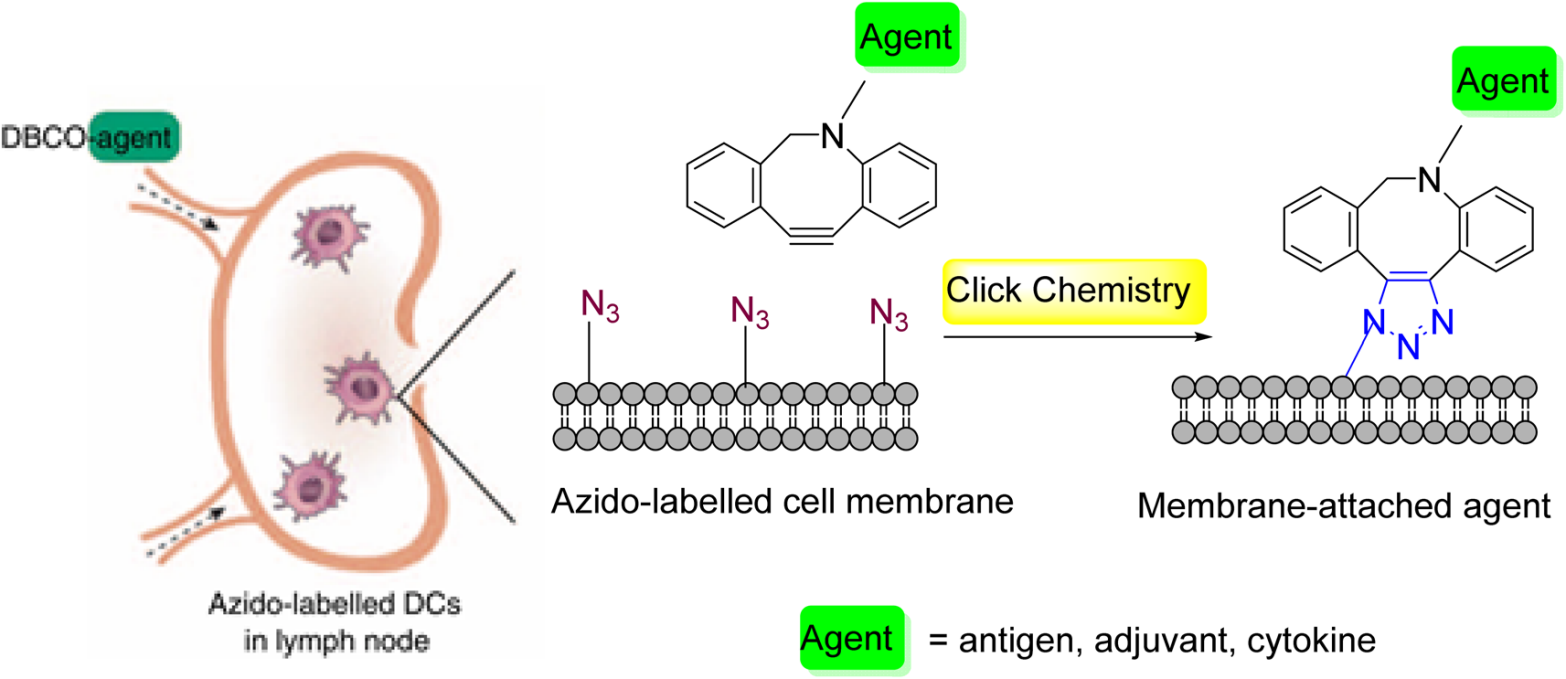
New strategy for the targeting of immunomodulatory agents *via* click chemistry.^26^

Wang *et al.*^26^ expanded this concept to *in vivo* cancer immunotherapy, demonstrating its feasibility in dendritic cells (DCs) (Fig. 4). They innovatively utilized an injectable pore-forming alginate gel to encapsulate azide-sugar molecules, with their release controlled by external ultrasound. Leveraging DC mobilization, functional azide motifs can be displayed on the membrane surface as needed, enhancing DC tracking. Furthermore, the efficient click chemistry of the azide-label supports the targeted delivery of DBCO-labeled tumor antigens, cytokines, adjuvants, and other immunomodulatory agents.

### 4.2. Bioorthogonal click chemistry in tracking cancer drugs

**Fig. 5:**
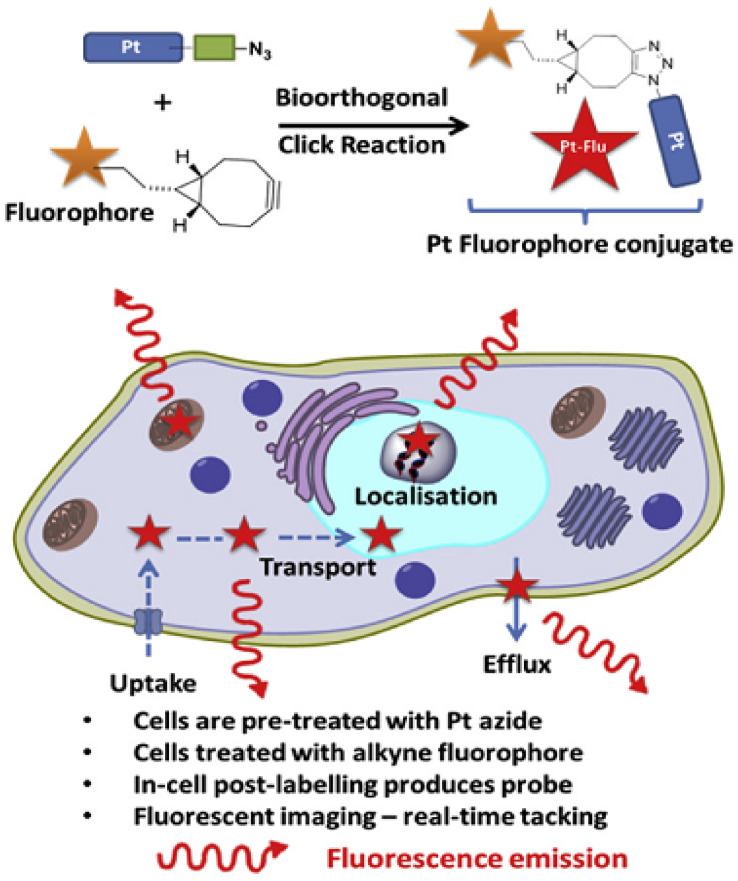
Strategy for click-enabled real-time tracking of Pt drug surrogates.^17^

Utilizing confocal microscopy for fluorescent detection of platinum drugs in cells enables researchers to monitor drug movement, providing crucial insights into cellular uptake, transport, localization within subcellular structures or organelles, and efflux. This fluorescent tracking can be achieved through post-binding bio-orthogonal ligation techniques within cells, as illustrated in Fig. 5.^17^ Platinum-based drug analogs with minimally disruptive bio-orthogonal click handles attached to stable amine carrier ligands can maintain the fundamental chemical and biological characteristics of the original drug.

The application of click chemistry methods (CuAAC or SPAAC) has proven effective for mapping the subcellular distribution of post-labeled platinum drug surrogates in fixed cancer cells, including cell cycle-specific localization. However, future advancements in tracking platinum-based drugs are expected to focus on real-time monitoring of platinum click templates in living human cancer cells using copper-free click reactions that trigger fluorescence activation. Challenges in this area include the solubility of SPAAC-based fluorophores and ensuring the stability and non-interference of click handles.^17^

Approximately half of cancer patients undergoing chemotherapy receive a Pt(ii)-based drug such as cisplatin, carboplatin, or oxaliplatin.^17^ Cisplatin and carboplatin primarily function by forming cross-links with nuclear DNA; these Pt-DNA complexes disrupt transcription, trigger DNA damage responses, and eventually lead to apoptosis. Indeed, the clinical efficacy of Pt anticancer agents is limited by toxic side effects and both inherent and developed resistance.^27^ To aid this endeavor, the development of innovative techniques for synthesizing, labeling, and tracking Pt(ii)- and Pt(iv)-based complexes is significantly needed. For this goal, click chemistry served as a versatile and powerful tool that could be utilized for the development of novel Pt-based anticancer drugs by functionalizing them with biomolecules to enhance tumor targeting.^17^ This has led to improved understanding of their biological effects at the cellular level. Organic click reactions between azides and alkynes have been employed to modify the ligands of platinum complexes, incorporating targeting agents and fluorophores.

This approach enables the attachment of various elements such as targeting agents, delivery systems, fluorescent markers, and secondary chemotherapeutic molecules.^17^

## 5. Signature of click chemistry in advanced techniques for cancer therapeutics

### 5.1. Click chemistry in the synthesis of antibody–drug conjugates

An antibody–drug conjugate (ADC) features a monoclonal antibody (mAb) chemically linked to a cytotoxic agent *via* a covalent attachment. This combination leverages the precise targeting capabilities of antibodies with the potent cell-killing effects of drugs to effectively eliminate cancer cells. The antibody component binds to specific antigens on tumor cells, facilitating targeted drug delivery. As a result, ADCs offer the therapeutic benefits of chemotherapy while reducing systemic toxicity.^28^

ADCs have emerged as a hotspot in cancer drug development. Since the FDA's approval of Mylotarg® (gemtuzumab ozogamicin) in 2000, 14 ADCs have received market authorization globally. Currently, more than 100 ADC candidates are undergoing clinical trials. These novel anti-cancer therapeutics, often referred to as “biological missiles,” are ushering in a new era of targeted cancer treatment.^29^

**Fig. 6:**
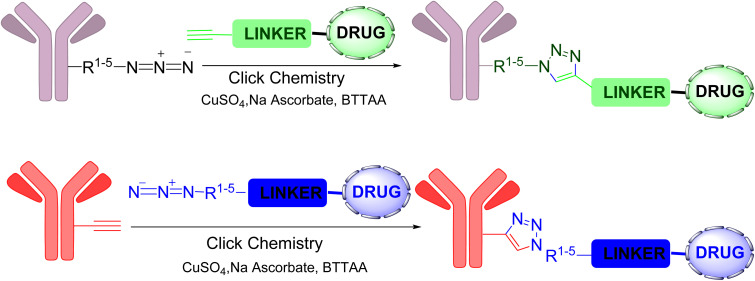
Synthesis of novel antibody–drug conjugates (ADCs).^28^

Vatansever and colleagues^28^ explored the creation of new ADCs using a CuAAC reaction between a metal-chelating azide attached to the drug and an alkyne integrated into the antibody (Fig. 6). They reported that the metal-chelating azide incorporated into the antibody requires specific reaction conditions, potentially leading to increased heterogeneity in the synthesized ADCs. Such heterogeneity can result in reduced activity and possible toxicity. In contrast, alkyne-incorporated antibodies do not have this limitation and are more accessible and cost-effective to produce. To validate their hypothesis, the researchers used Pertuzumab as a model antibody. They reduced it and then incorporated alkyne and 2-azidopyridine linked to the cytotoxic payload. The payload-antibody linkage was efficiently achieved under the standard click reaction conditions and the entire ADC synthesis process furnished excellent yield in short time, confirming that the CuAAC reaction is suitable for rapid and efficient ADC production.^28,30^

**Fig. 7:**
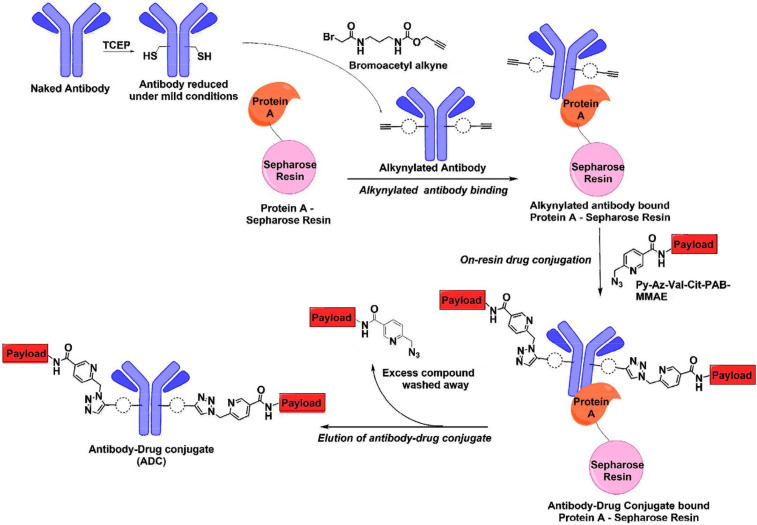
Catch-and-release synthesis of antibody–drug conjugate (ADC).^31^

Vatansever *et al.*^31^ illustrated the benefits of combining an antibody alkyne with a metal-chelating drug azide by developing a catch-and-release synthesis method,^31^ as depicted in Fig. 7. This approach allows for the use of a bromoacetyl alkyne to quickly produce an alkyne-modified antibody, with any remaining alkyne compound easily removed through washing. The resulting antibody alkyne can then be efficiently combined with a metal-chelating drug azide to create the ADC. This process significantly reduces production expenses and minimizes the generation of toxic waste materials. The authors used Pertuzumab, a monoclonal antibody utilized in treating metastatic HER2-positive breast cancer. Pertuzumab-2C was first reduced under mild conditions followed by reaction with bromoacetyl alkyne to load the alkyne group. Subsequently, the antibody was incubated with protein A-Sepharose for 2 h to capture the alkynylated antibody on the resin. The residual alkyne compound was subsequently washed out, providing AzPy-payload per alkynylated cysteine. The reaction ends with washing out the residual AzPy-payload and eluting the conjugated Pertuzumab.^31^

**Scheme 1:**
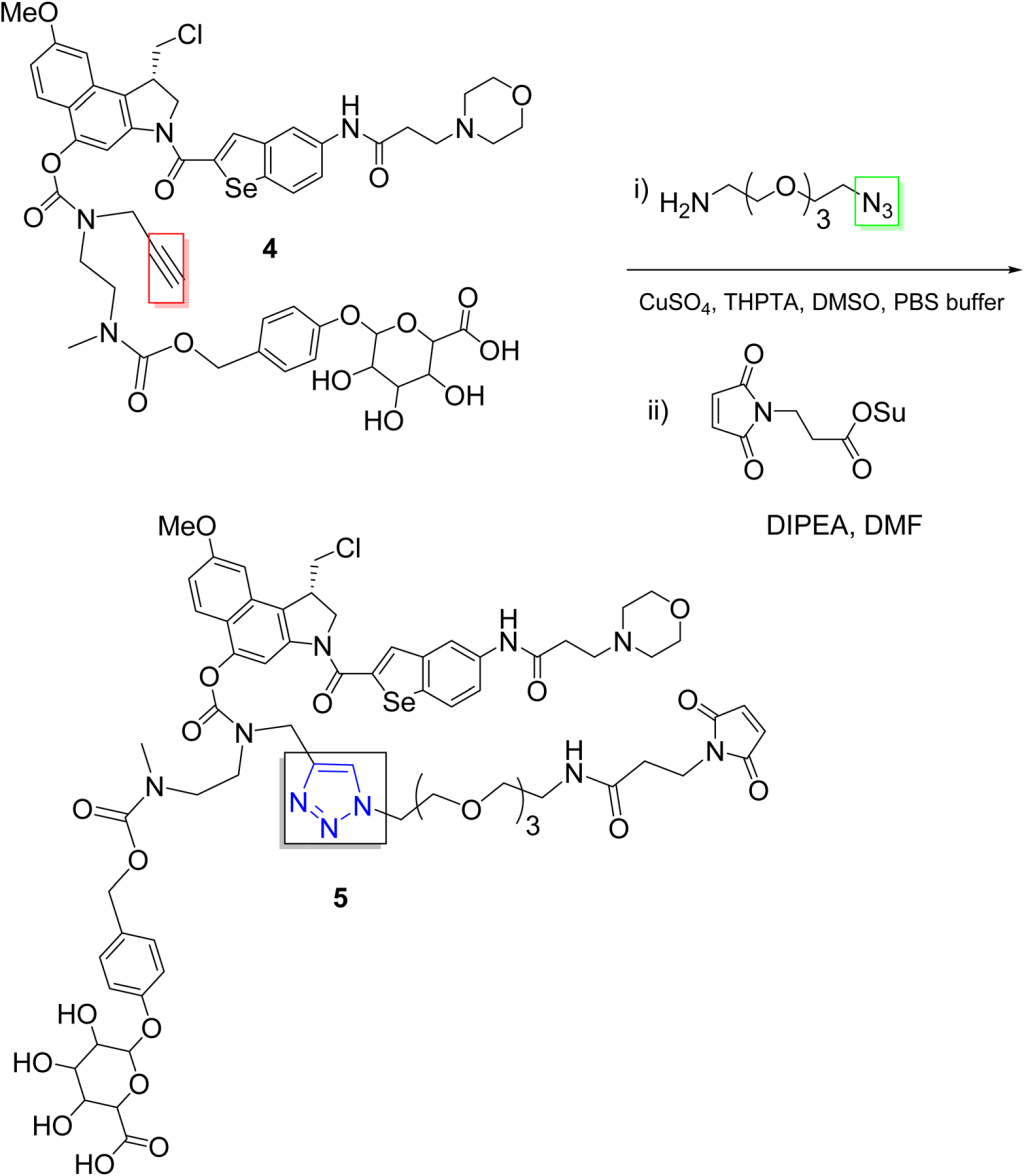
Click approach for the development of ADCs with hydrophilic linkers.

One major challenge in ADC development is aggregation, due to the poor aqueous solubility of drug payloads. Recent advances have addressed this issue using click chemistry (Scheme 1). For example, Lim and co-workers developed hydrophilic linkers for drug payloads *via* PEGylation with click chemistry, which enabled the successful development of ADCs for the duocarmycin class of compounds, known for their potent antitumor activity.^32^

### 5.2. Click chemistry in the development of PROTACs

Proteolysis-targeting chimeras, abbreviated as PROTACs, are typically hetero-bifunctional molecules that were designed to facilitate target protein disposal by recruiting the ubiquitination-proteasome degradation machinery. Indeed, the chimeric nature of these molecules necessitates an “assembling” step, either in the lab or *in situ*, during synthesis. Targeted PROTACs often require a second “assembling” step due to their hetero-trifunctional nature. Click chemistry offers unique advantages in connecting two or more molecular entities efficiently under near-physiological conditions, making it applicable to PROTAC development in various ways.^33^

**Scheme 2:**
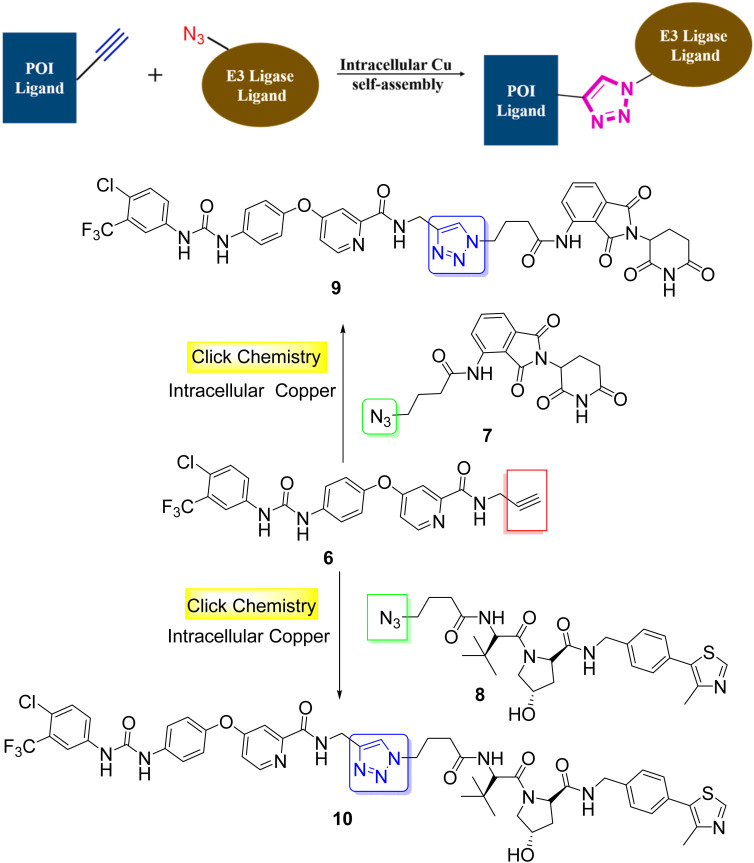
Synthesis of anti-cancer PROTACs *via* CuAAC reaction *in vivo*.

Si *et al.* proposed an interesting example of anti-cancer PROTACs with high cell permeability and selectivity, based on a click reaction (CuAAC) *in vivo* catalyzed by endogenous Cu (Scheme 2).^34^ Sorafenib, a multi-targeted kinase inhibitor, as a POI ligand was transformed into the alkyne terminated precursor 6. Azide-terminated ligands 7 and 8 were used as E3 ligase binders. The increased level of endogenous Cu in tumor cells catalyzed the *in situ* self-assembly of the two clickable precursors, generating PROTACs 9 and 10.^34^

**Scheme 3:**
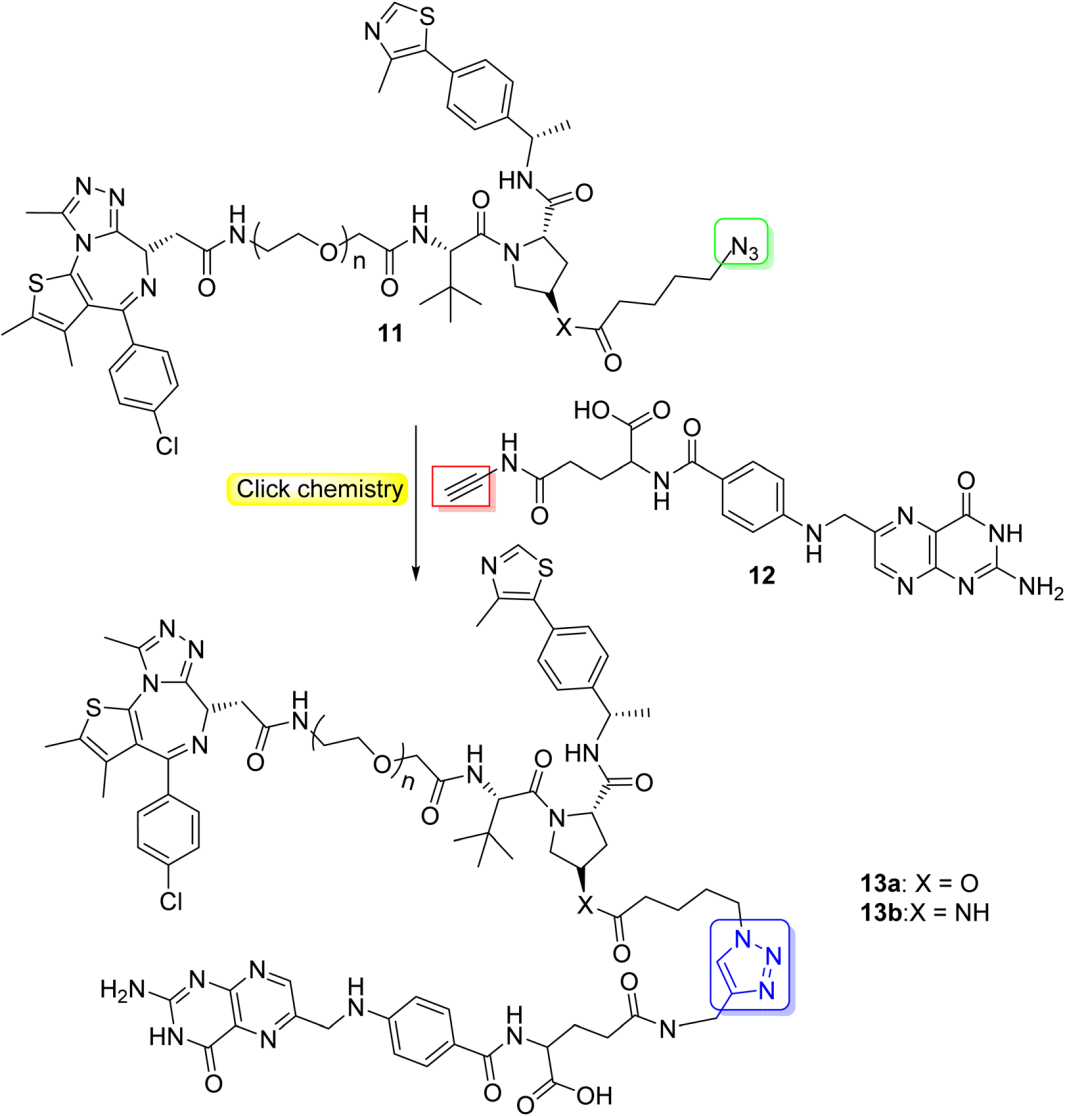
Click synthesis of folate conjugated PROTACs.

In addition to antibody-mediated targeted delivery, small molecule vectors can also be employed. Folate receptors, which are overexpressed in various cancer types such as lung-, ovarian-, breast-, and colorectal cancers, provide an opportunity for targeted drug delivery using folate-conjugated drugs. The folate component acts as a ligand that binds in a selective manner to cancer cells with high folate receptor expression, enabling targeted drug delivery to tumor sites. Indeed, folate conjugation presents chemical challenges which could be addressed by click chemistry.^35^ The high expression of folate receptors in many cancer cell types allows for selective delivery of PROTACs to these cells. Liu *et al.* utilized click chemistry (CuAAC) to synthesize folate-conjugated PROTAC 13a through conjugation of the azido-functionalized PROTAC molecule 11 with the folate-alkyne 12 (Scheme 3).^36^ Various cancer cell lines with high folate receptor expression underwent treatment with folate-conjugated 13a at different concentrations. After incubation, significant degradation of the BRD4 oncoprotein was observed in human cervix carcinoma (HeLa) cells. Therefore, by leveraging folate receptor-mediated internalization, folate-conjugated PROTACs can enhance the tissue selectivity of PROTACs towards cancer cells and at the same time minimize the toxicity and side effects in normal tissues and cells.^36^

**Fig. 8:**
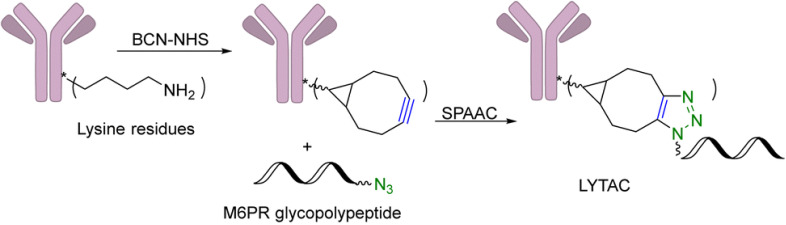
Construction of a LYTAC using SPAAC for degradation of EGFR in HeLa cells.^37^

In an effort to broaden the use of PROTAC-like molecules, Bertozzi and her group have introduced lysosome-targeting chimeras (LYTACs) for degradation of extracellular proteins.^37^ Initial studies demonstrated this concept by connecting a protein-targeting component to a glycopeptide ligand, which interacts with and stimulates a cell-surface receptor that shuttles to lysosomes, using SPAAC. The specific target in these experiments was anti-mouse IgG. As illustrated in Fig. 8, the process involves non-specific labeling of lysine residues in polyclonal anti-mouse IgG with bicyclononyne-*N*-hydroxysuccinimide (BCN-NHS), followed by SPAAC-mediated conjugation to an azide-terminated glycopolypeptide. This glycopolypeptide binds to a cation-independent mannose-6-phosphate receptor (CI-M6PR). In essence, these dual-function lysosome-targeting chimeras can degrade proteins outside the cell, thus overcoming the constraint of PROTACs, which are limited to intracellular protein targets.^37^

### 5.3. Application of click chemistry in modification of extracellular vesicles for cancer therapy

Extracellular vesicles (EVs), small vesicles released by cells into the extracellular space, have been found in almost all body fluids.^38^ They have emerged as promising therapeutic vehicles due to their innate ability to transport biomolecules and cross biological barriers. However, their limited targeting precision and cargo capacity requires modifications to enhance therapeutic efficacy.^38^ EVs can be broadly classified into exosomes, microvesicles, and apoptotic bodies based on their biogenesis, size, and molecular composition. Click chemistry presents an innovative approach to modifying EV surfaces, allowing the precise attachment of targeting ligands, imaging agents, and therapeutic molecules, thereby improving the targeting, delivery, and overall effectiveness of EV-based therapies.^39^

Ciferri and coworkers succeeded in modifying extracellular vesicle (EV) membranes using a fluorescent azide as a reporter molecule in a two-step copper-free click chemistry reaction.^40^ This approach enabled easy and reliable detection of engineered EVs, streamlining the optimization and quality assurance of the membrane modification process. The modified EVs demonstrated effective time-dependent uptake by target tumor cells, with partial recycling occurring in the cells' endosomal compartment. The protocols established in this study provide a solid foundation for future research, where more specialized molecules could be utilized to confer specific functionalities to EV membranes.

**Fig. 9:**
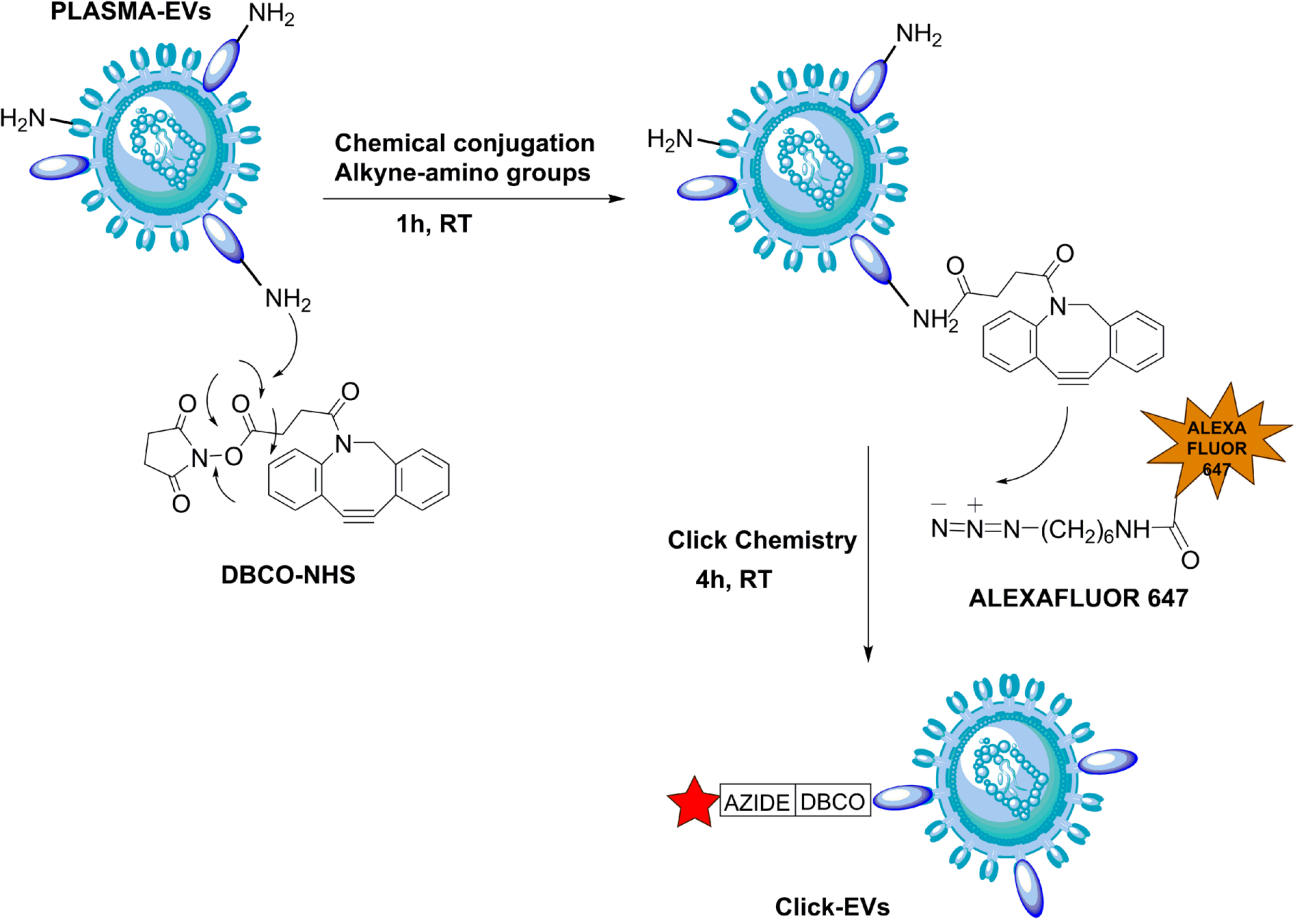
Functionalization of the plasma-EV surface through a copper-free click chemistry strategy.^40^

The plasma-EV membrane has been modified using a copper-free click chemistry method. This technique employs dibenzocyclooctyne-*N*-hydroxysuccinimidyl ester (DBCO-NHS) as an alkyne reagent and a fluorescently tagged azide (AZ-647) as a model molecule to establish the protocol (Fig. 9). Initially, the amino groups on EV surface proteins interact with the *N*-hydroxysuccinimidyl (NHS) component of DBCO. Subsequently, the alkyne part of DBCO binds to AZ-647 through copper-free click chemistry. The optimal labeling efficiency was achieved when EVs are exposed to DBCO-NHS for 1 hour at room temperature, followed by immediate coupling with AZ-647 for 4 hours at room temperature.^40^

**Fig. 10:**
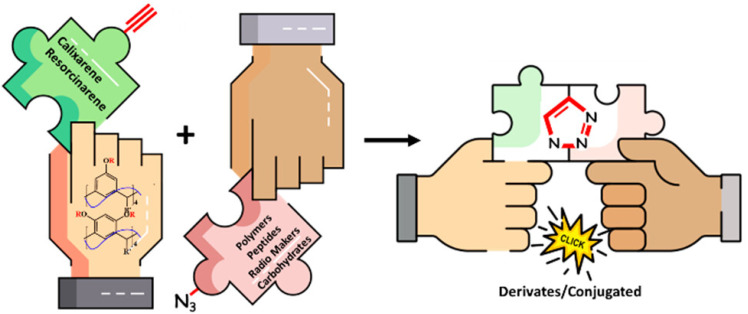
Click chemistry in modification of exosomes for cancer therapy.^41^

Exosomes are an important kind of extracellular vesicle (EV) that are found in almost all body fluids. Exosomes modified through click chemistry offer a promising approach to tackle cancer heterogeneity by targeting various cancer cell populations within tumors. This method enhances treatment precision and helps combat drug resistance (Fig. 10). The introduction of SPAAC reduces toxicity concerns, ensuring the approach is both biocompatible and safe. As scientific investigations progress, this strategy demonstrates significant potential for tailored and efficient cancer therapies, laying the groundwork for innovative diagnostic tools and treatment options in the future.^41^

A technique utilizing click chemistry was developed to attach ligands to exosome surfaces. The CuAAC reaction is particularly suitable for this purpose due to its rapid reaction time, high specificity, and compatibility with aqueous environments. This method of conjugation does not significantly alter exosome size or affect their ability to attach to and enter recipient cells. Exosomal proteins can be easily modified with alkyne groups, which can then bind to azide-containing molecules through cycloaddition reactions. This approach has been employed to modify exosome surfaces with both small dyes and larger azide-containing proteins.^42^

### 5.4. Click chemistry in photodynamic therapy (PDT)

Photodynamic therapy (PDT) is a promising anticancer modality that uses a photosensitizer (PS) to generate destructive reactive oxygen species (ROS), particularly singlet oxygen ^1^O_2_, upon irradiation at an appropriate wavelength. These ROS eliminate unwanted cells (particularly cancer cells) through apoptosis. PDT offers a selective and minimally invasive therapeutic option capable of inactivating pathogenic cells without causing harm to surrounding healthy tissues.^43–45^

**Scheme 4:**
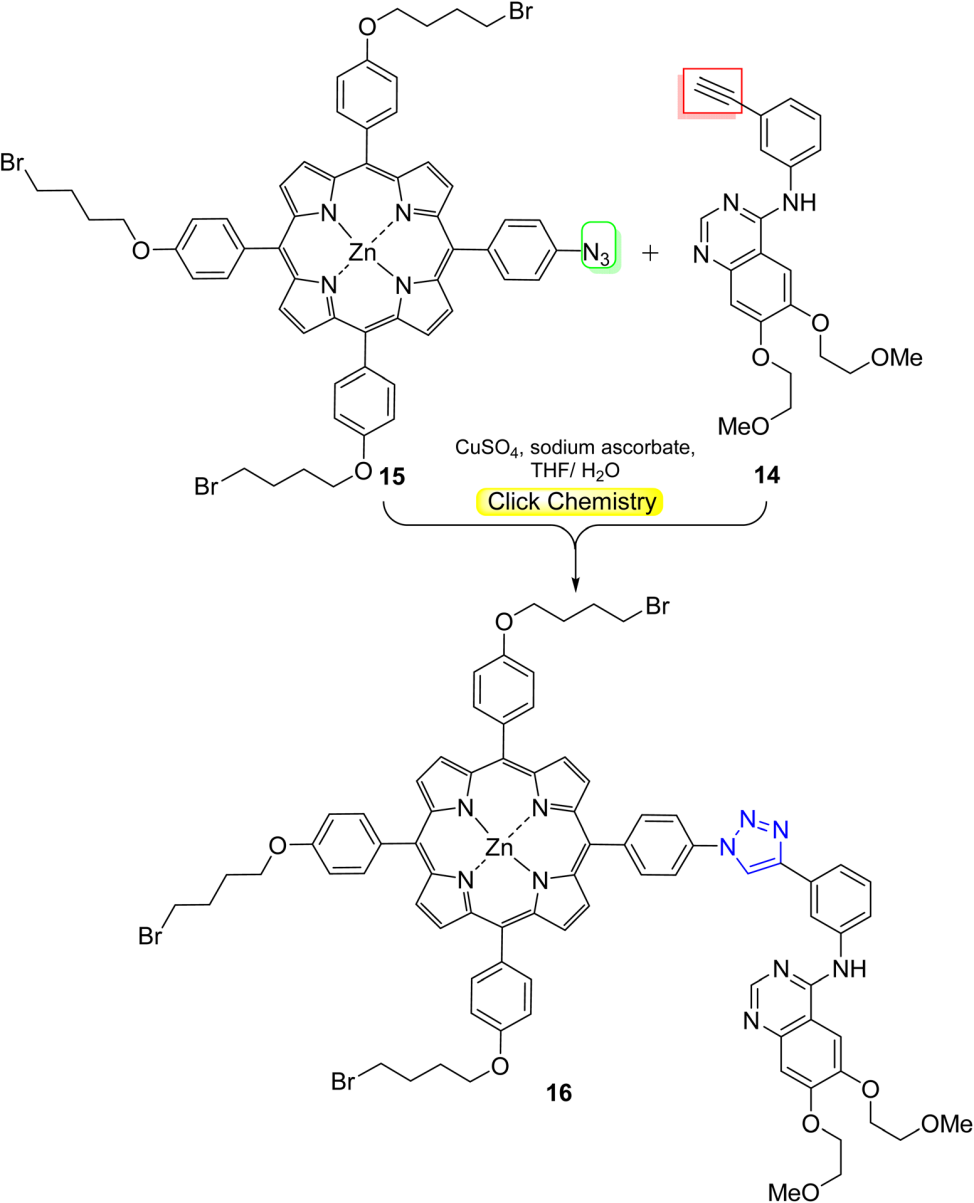
Click synthesis of erlotinib–porphyrin conjugate 16 as PDT agent.

*Meso*-tetrasubstituted porphyrins^46^ are considered the most popular porphyrin-based molecules developed as effective PSs for PDT. Novikov and coworkers^47^ designed a PDT agent based on *meso*-arylporphyrin with tyrosine kinase inhibitor (erlotinib) through a click reaction of erlotinib–alkyne 14 with azidoporphyrin 15, then pyridine quaternization furnished the cationic erlotinib–porphyrin conjugate 16 (Scheme 4). They also prepared and characterized a nano-formulation based on Pluronic F127 micelles. The results exerted a significant difference between the dark and photoinduced activity of 20–40-fold for the conjugate nanomicelles. Upon irradiation, 1.8 times more of toxicity was recorded for the conjugate nanomicelles against the EGFR-overexpressing cell line MDA-MB-231 (IC_50_ = 0.073 μM) relative to the normal NKE cells (IC_50_ = 0.13 μM).

**Scheme 5:**
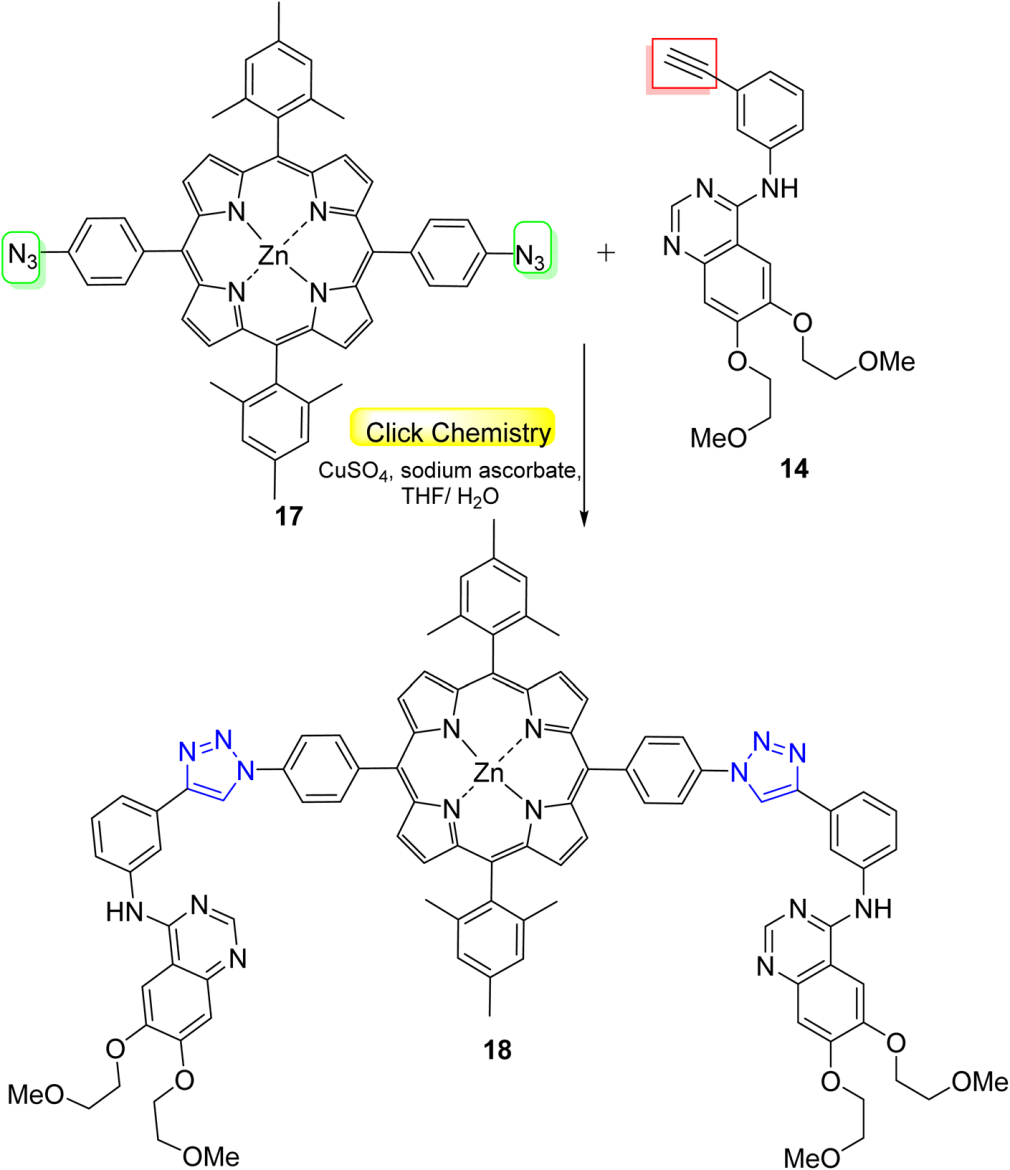
Synthesis of porphyrin-based conjugate containing two erlotinib molecules as a PDT agent *via* the ‘double-click’ reaction.

**Fig. 11:**
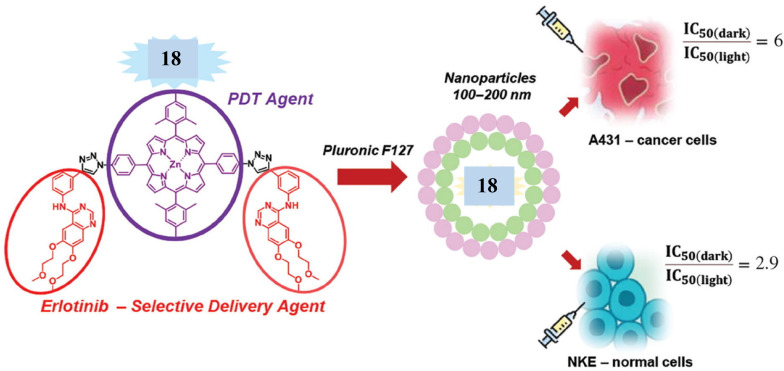
New cytotoxic conjugate based on *meso*-arylporphyrin and erlotinib.^48^

In a similar manner, Zhdanova *et al.*^48^ synthesized phototherapeutic agent using a click reaction between bis(4-azidophenyl)-porphyrin 17 and erlotinib 14 (Scheme 5). The new hybrid 18 was examined as a potential PS for targeted PDT, and the results revealed that the conjugate recorded excellent photoinduced cytotoxicity (IC_50_ = 0.86 and 0.54 μm) for NKE and A431 cells, respectively (Fig. 11).

**Fig. 12:**
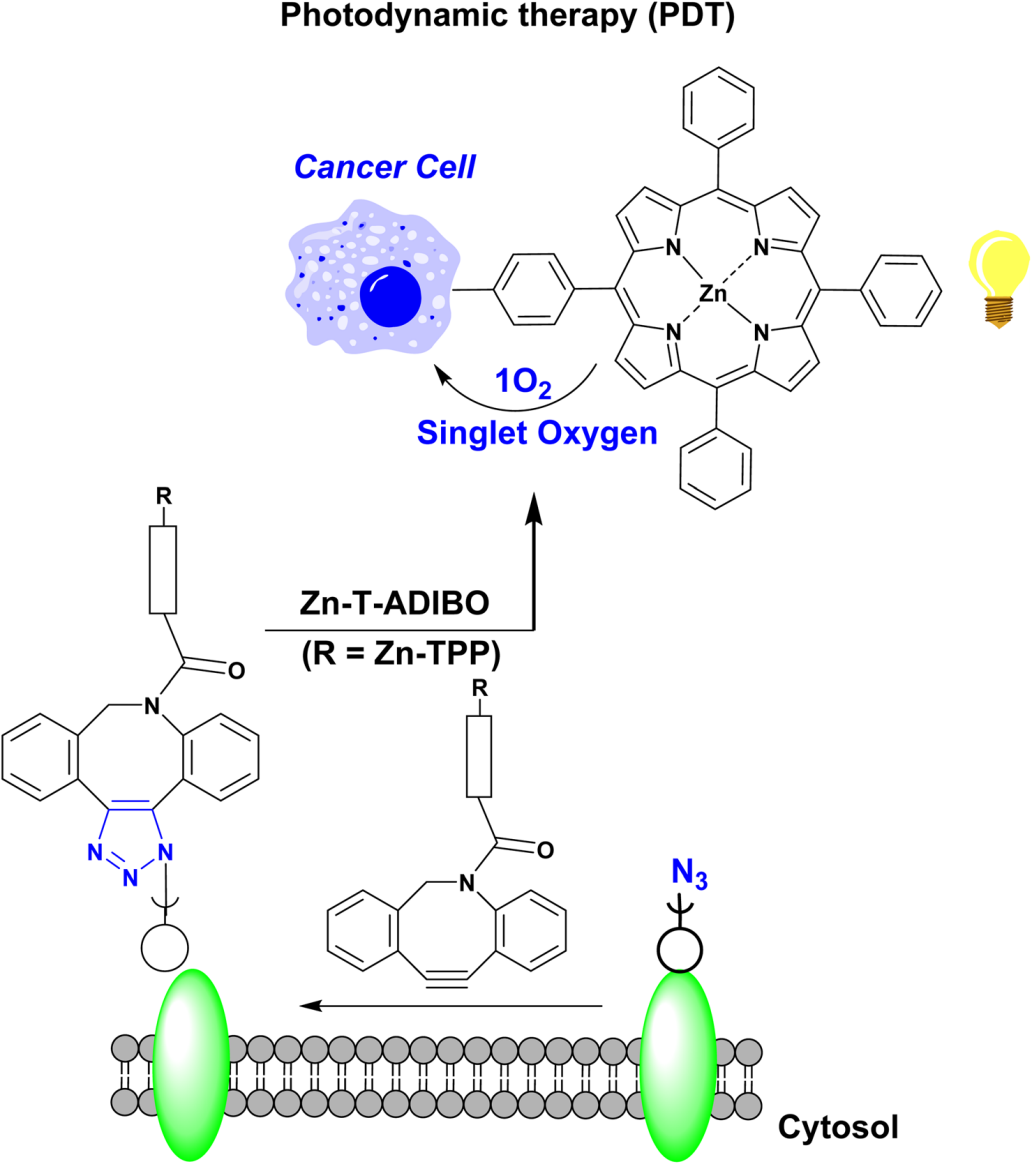
Strategy for promoting cancer cell death using metabolic glycan labelling coupled with PDT.^49^

Park *et al.*^49^ developed a strategy to induce cancer cell death using PDT,^49^ where the cell-surface azide-containing glycans were labelled with Zn-T-ADIBO featuring azadi-benzocyclooctyne (ADIBO) and Zn-tetraphenylporphyrin (Zn-TPP) (Fig. 12). Light irradiation of PS-modified cancer cells led to the production of ROS, particularly (^1^O_2_), that caused the apoptosis of target cancer cells. The study aimed to assess the real impact of PDT on inducing cancer cell death in cells incubated with a glycan precursor. Human colorectal adenocarcinoma cell line (HT-29), human colon carcinoma (HCT116), and human breast adenocarcinoma (MCF-7) were used as models for human cancers. After pretreatment with H_2_O_2_, these cells were exposed to PBA-ManNAz(OAc)_3_ and Zn-T-ADIBO sequentially to label azide-containing glycans on the cell surface with a PS. The treated cells were then subjected to light exposure and incubated for one day. LDH assay results revealed that cancer cell death was significantly increased when all components were applied and followed by light exposure. However, cell death did not occur when any single component or the irradiation step was discarded from the protocol.^49^

**Scheme 6:**
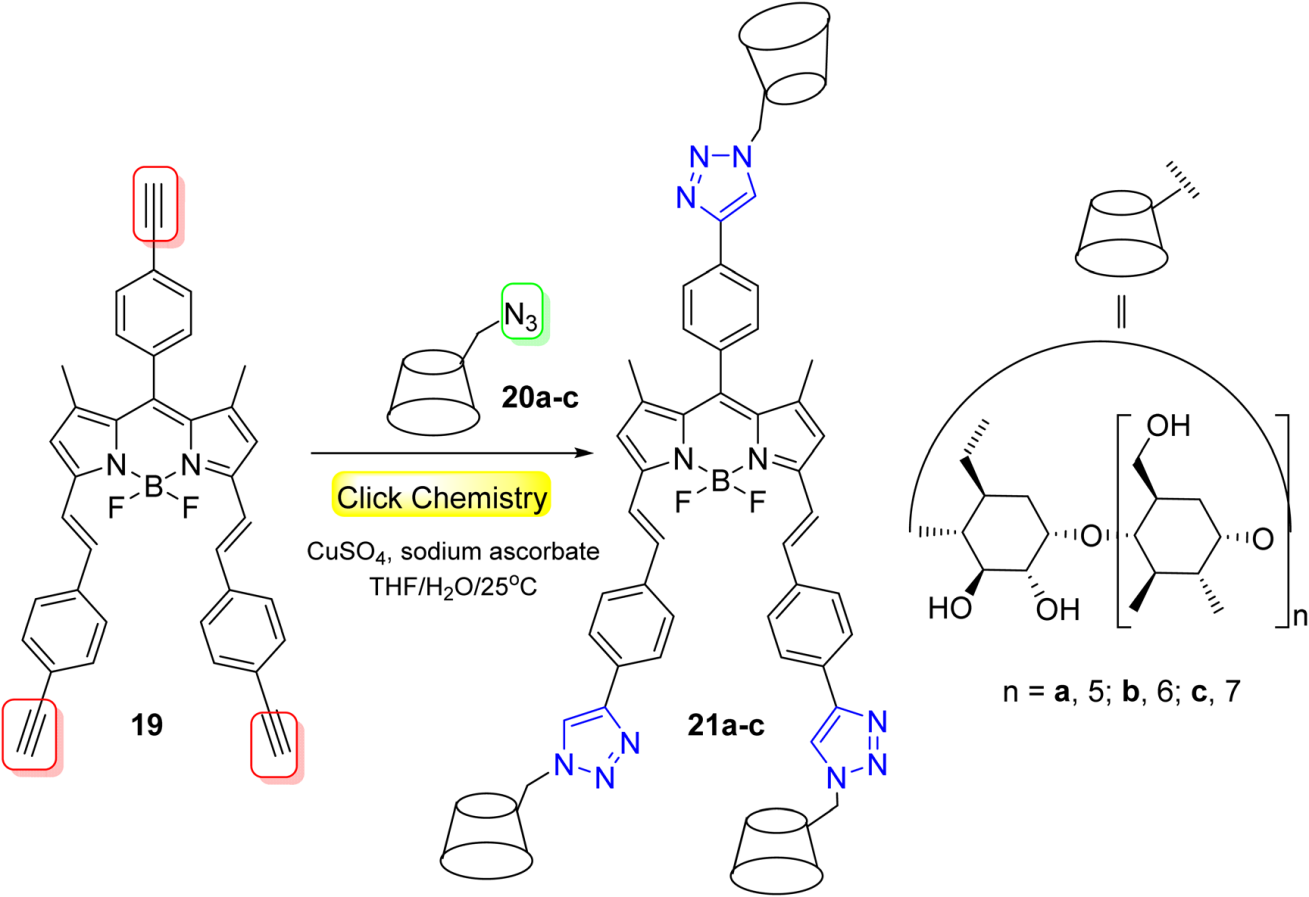
Click synthesis of BODIPY-CD complexes as PDT agents.

BODIPY-cyclodextrin 21a–c were designed to serve as PDT agents by Lu *et al.*^50^ through the click reaction of BODIPY-alkynyl 19 and cyclodextrin-azide 20a–c (Scheme 6). The BODIPY-CDs 21a–c were found to have superior water solubility owing to the existence of CD, and their fluorescence emissions were clearly redshifted by more than 90 nm. The BODIPY-CDs 21a–c were not cytotoxic toward NIH 3T3 at different drug concentrations (which was anticipated to be utilized in the bio-imaging technology), while they exhibited good inhibition on tumor HeLa cells. In particular, BODIPY-β-CD 21b generated high ROS with considerable PDT activity against HeLa cells.

**Scheme 7:**
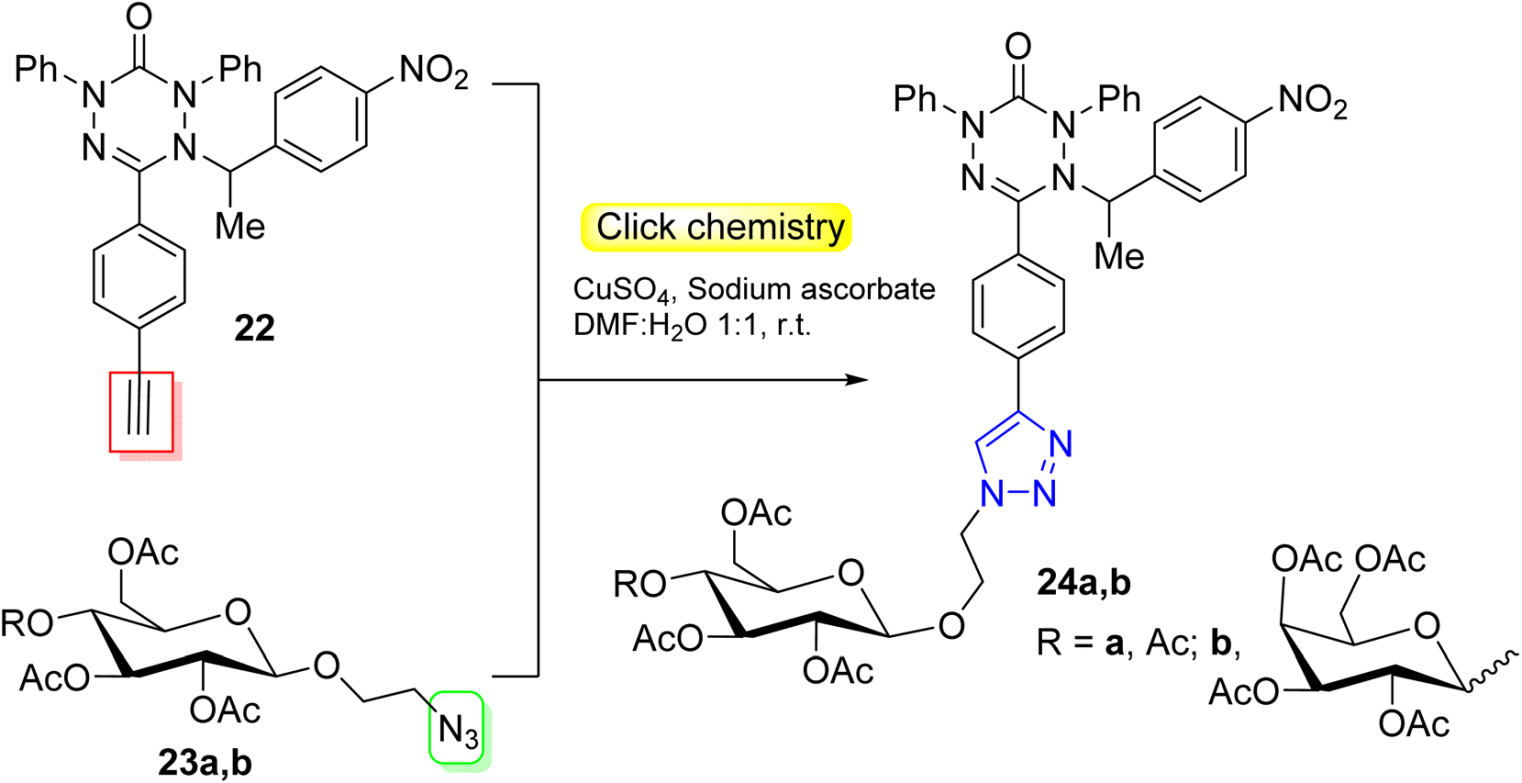
Click synthesis of sweetened alkylated verdazyls as PDT agents.

Apart from the pyrrole photosensitizers (BODIPYs and porphyrins), Votkina *et al.*^51^ described an effective example for killing cancer cells under light irradiation through the preparation of sweetened alkylated verdazyls (Scheme 7).

Conjugates of carbohydrates with 1,2,4,5-tetrazin-3(2*H*)-one derivative (AlkVZs) 24a,b were obtained *via* a click reaction of tetrazin-alkyne 22 with sugar-azide 23a,b. The produced conjugate exhibited high oxygen-independent cytotoxicity on the cancer cells (PC-3 and Jurkat) under light irradiation and at the same time, they exert low toxicity in the dark. MTT and Alamar Blue tests were used to estimate the efficacy of prepared conjugates, in addition to the microscopic dead/live colored images and flow cytometry.

### 5.5. Click chemistry in targeted dual-agent combination therapy for cancer

**Fig. 13:**
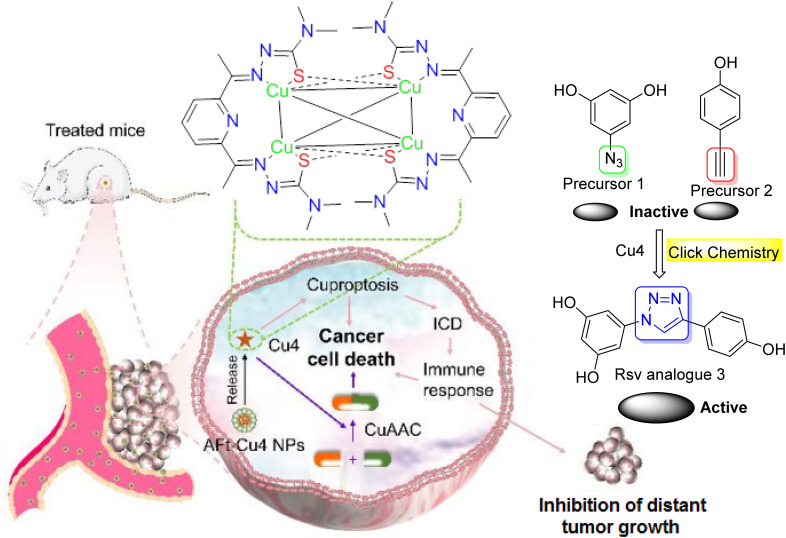
Dual-agent strategy based on tetranuclear Cu(i) complex and Aft.

Click chemistry could also contribute to the targeted dual-agent combination therapy for cancer. Recently, Man *et al.*^52^ reported the synthesis of a copper (Cu) complex for next-generation metal-based drugs and dual-drug combination cancer therapy.^52^ This complex was designed to catalyze a click reaction to produce a chemotherapeutic agent *in situ*, enabling targeted dual-agent combination chemotherapy and immunotherapy (Fig. 13). They also developed an apoferritin (AFt)-Cu_4_ nanoparticle (NP) delivery system. AFt-Cu_4_ NPs demonstrated enhanced tumor growth inhibition, along with improved targeting, and minimized systemic toxicity of Cu_4_*in vivo*. Notably, combining AFt-Cu_4_ NPs with the resveratrol analogue produced from the *in situ* CuAAC reaction amplified the anticancer effect. They additionally discovered that both cuproptosis and cuproptosis-induced systemic immune responses were involved in the anticancer mechanism of Cu_4_/AFt-Cu_4_ NPs.^52^

## 6. Conclusion, challenges, and future perspectives

The concept of ‘click chemistry’, introduced by Nobel Prize winner K. Barry Sharpless in 2001 and 2022 encompasses a group of highly efficient and selective chemical reactions. These processes allow for the swift and dependable creation of molecular structures (simple and complex). Known for their high yields, specificity, and modularity, click reactions have found applications in various fields, including drug discovery, bioconjugation, and material design. Since its inception, click chemistry has rapidly and significantly influenced numerous areas of modern chemistry, with research and development in this field continuing to grow exponentially.

Looking ahead, it is crucial to acknowledge the layers of obstacles that face click chemistry and its advanced strategies in achieving success during *in vivo* studies and their applications in cancer therapy.

For the next generation of ADC, crucial factors appear to be discovering and confirming novel antigen/antibodies, creating new payloads with ideal toxicity levels, and engineering innovative linkers that strike a balance between stability and payload release. ADC activation with “click-to-release” chemistry on a tumor cell represents a promising future perspective. This approach has shown significant potential, demonstrating high ADC uptake by tumors, precise chemical control of activation, and effective retention of the released model drug within the tumor. These findings establish a solid foundation for further research into therapeutic applications.

For exosomes: with ongoing advancements in research and clinical trials, click-modified exosomes are anticipated to become increasingly significant in the development of next-generation cancer therapeutics and diagnostics. These innovative exosomes show great potential for personalized and effective cancer treatments by addressing challenges related to specificity of targeting, capacity of therapeutic payload, in addition to the toxicity issues.

Through the application of copper-free click chemistry, the engineering of plasma extracellular vesicle membranes provides a promising approach for tailoring exosomes to specific therapeutic or diagnostic applications. By precisely functionalizing the exosome membrane, it is possible to enhance their stability, targeting efficiency, and cargo delivery capabilities, thereby unlocking the full potential of exosomes as versatile and effective drug delivery vehicles or diagnostic tools.

For PDT: significant research should be directed towards creating advanced photosensitizers capable of improved tumor targeting and regulated ROS production, with the aim of enhancing treatment effectiveness and reducing adverse effects. Owing to the high orthogonality, different click reactions can be used sequentially to introduce tumour-targeting ligands to PS's, deliver PS's to pre-labelled bio-objects and activate the photodynamic activity of PS's *via* bioorthogonal removal of the quenching unit or *in situ* synthesis of the PS's. These strategies could be applied to molecular and nano-based photosensitizing systems, resulting in targeted delivery and site-specific activation of these photo-responsive therapeutic agents, which can potentially actualize precise PDT.

For PROTACs: there are extra layers of challenges for the success of PROTACs in *in vivo* studies. To overcome the issues of high molecular weight and low permeability of PROTACs, *in situ* assembly using click chemistry has been successfully used. There is also the issue of off-tumour toxicity, which is a significant consideration in the development of PROTACs. Researchers are constantly exploring the use of click chemistry to link PROTACs with targeting molecules for precise delivery to cancer cells. A click-and-release strategy offers a targeted method. While PROTACs are limited to targeting proteins within cells, the potential application and viability of click chemistry for degrading extracellular and membrane-bound proteins in lysosomes presents a robust solution to address the limitations of PROTACs.

An additional hurdle in PROTAC development is the necessity to enhance the efficiency and quality of PROTAC libraries. For this purpose, click chemistry offers a versatile set of tools that significantly aids in expediting PROTAC discovery and refinement. This approach enables the rapid creation of PROTAC libraries and facilitates faster identification of effective degraders.

## List of abbreviations

A431 cells: Human epidermoid cancer cell line
A549: Human alveolar basal epithelial cells
ABPP: Activity-based protein profiling
ADC: Antibody–drug conjugate
ADIBO: Azadibenzocyclooctyne
AFt: Apoferritin
AlkVZs: Alkylated verdazyls
BCN: Bicyclononyne
BODIPY: Boron dipyrromethenes
BRD4: Bromodomain and extra terminal domain
BTTAA: 2-(4-((Bis((1-(*tert*-butyl)-1*H*-1,2,3-triazol-4-yl)methyl)amino)methyl)-1*H*-1,2,3-triazol-1-yl)acetic acid
CC_50_: Half maximum cytotoxic concentration
CD: Cyclodextrin
Cu: Copper
CuAAC: Copper(i)-catalyzed azide–alkyne cycloaddition
CuSO_4_: Copper sulfate
DBCO: Dibenzocyclooctyne
DCs: Dendritic cells
DIBAC: Aza-dibenzocyclooctyne
DIBO: Dibenzoannulated cyclooctyne
DNA: Deoxyribonucleic acid
E3: Ubiquitin ligase
EGFR: Epidermal growth factor receptor
EtOH: Ethanol
FDA: Food and Drug Administration
HCT-116: Human colon carcinoma
HeLa: Human cervix carcinoma
HT-1080: Human fibrosarcoma cell line
HT-29: Human colorectal adenocarcinoma cell line
IARC: International Agency for Research on Cancer
IC_50_: Half maximum inhibition concentration
K562: Erythroleukemia cell line
KB: Adherent epithelial cell line
L929: Mouse fibroblast cell line
LC3: Light chain 3
LDH: Lactate dehydrogenase test
mAb: Monoclonal antibody
MDA-MB-231: Triple-negative breast cancer
MeOH: Methanol
MTX: Methotrexate
*m*-THPC: *Meta*-tetra(hydroxyphenyl)chlorin
*m*-THPP: 5,10,15,20-Tetrakis(3-hydroxyphenyl)porphyrin
MTT: 3-(4,5-Dimethylthiazol-2-yl)-2,5-diphenyltetrazolium bromide
NKE: Human normal kidney epithelial cell line
NPs: Nanoparticles
NSCLC: Non-small cell lung cancer
PC-3: Human prostate cancer cell line
POI: Protein of interest
PROTACs: Proteolysis-targeting chimeras
PS: Photosensitizer
Pt: Platinum
RNA: Ribonucleic acid
ROS: Reactive oxygen species
SI: Selective index
SPAAC: Strain-promoted [3 + 2] azide–alkyne cycloaddition
*t*-BuOH: *Tert*-butanol
TPP: Tetraphenylporphyrin
VEGFR: Vascular endothelial growth factor receptor
Zn: Zinc

## Data availability

No primary research results, software or code have been included and no new data were generated or analysed as part of this review.

## Conflicts of interest

The authors declare no conflict of interest.
